# Changes in cell proliferation, but not in vascularisation are characteristic for human endometrium in different reproductive failures - a pilot study

**DOI:** 10.1186/1477-7827-8-67

**Published:** 2010-06-21

**Authors:** Ariane Germeyer, Michael von Wolff, Julia Jauckus, Thomas Strowitzki, Tanuj Sharma, Anna T Grazul-Bilska

**Affiliations:** 1 Department of Gynecological Endocrinology and Reproductive Medicine, University Hospital Heidelberg, Heidelberg, Germany; 2 Department of Gynecological Endocrinology and Reproductive Medicine, University Hospital Berne, Berne, Switzerland; 3 Department of Animal Sciences and Cell Biology Center, North Dakota State University, Fargo, North Dakota, USA

## Abstract

**Background:**

Reproductive failure, determined as recurrent spontaneous abortions (RSA) or recurrent implantation failure (RIF) in women is not well understood. Several factors, including embryo quality, and cellular and molecular changes in endometrium may contribute to the insufficient feto-maternal interaction resulting in reproductive failure. Prior clinical studies suggest an inadequate endometrial growth and development of the endometrium, leading to a lesser endometrial thickness.

**Methods:**

We therefore aimed to determine the cellular proliferation using Ki67, and the expression of markers of vascularisation, such as factor VIII (a marker of endothelial cells) and smooth muscle cell actin (SMCA; a marker of pericytes and smooth muscle cells) in endometrium of healthy women and women with RSA or RIF. LH-dated mid-secretory endometrial biopsies of seven healthy women and twenty women with reproductive failure were examined via immunohistochemistry followed by image analysis.

**Results:**

Cellular proliferation but not expression of factor VIII or SMCA was decreased (P < 0.0004) in endometrium of women with RSA and RIF compared to healthy controls. Conclusion: Our data indicate that reproductive failure is due to insufficient cell proliferation/tissue growth rather than inadequate vascularisation in the endometrium.

## Background

Reproductive failure comprises idiopathic recurrent spontaneous abortions (RSA) as well as recurrent implantation failure (RIF) [1]. One of the current RIF definition is a failure to conceive after three transfers of one or more good quality embryos; however, this definition may vary depending on the center [2]. On the other hand, idiopathic RSA is defined as three or more consecutive pregnancy losses within the first 20 weeks of gestation after exclusion of known contributing factors including uterine malformations, bleeding abnormalities, hormone disequilibrium, parental chromosomal defects, infections and others [3].

Adequate implantation is a limiting factor in human reproduction. Despite major improvement in assisted reproduction techniques (ART) the clinical pregnancy rate per embryo transfer in fresh ART cycles is 31%, and up to 41% in oocyte donation programs [4]. A number of etiologies, including decreased embryonic quality, endometrial receptivity, feto-maternal communication, endocrine, genetic and other factors have been suggested as causes for reproductive failure [5-7]. Even though intrauterine endometrium is not essential for embryo implantation, since ectopic pregnancies occur [8], several studies indicated an altered endometrial function in women with reproductive failure [9,10]. Changes at the cellular and molecular level in endometrium from women with reproductive failure have been reported [8,11,12]; suggesting an impact of specific genes on the success of implantation. However, these studies determine mostly gene expression profiles and only partially examine the transcribed products.

Although inadequate regulation of endometrial growth has been recognized as a possible factor in reproductive failures [11,13-15], limited data is available concerning endometrial cell proliferation and its regulation. Furthermore, inconsistent results were obtained from studies of the role of endometrial vascularisation, angiogenesis, blood flow and/or endometrial thickness in women with implantation failure; while some studies demonstrate differences between normal and reproductive failures subjects others do not [16-19].

We hypothesized that reproductive failures are due to altered uterine growth caused by changes in cell proliferation and vascularisation/angiogenesis in the endometrium. Therefore, the aim of this study was to examine cellular proliferation and expression of markers of vascularisation (factor VIII and smooth muscle cell actin [SMCA]) at the protein level in endometrial biopsies from the mid-secretory phase of healthy women and women with reproductive failure.

## Methods

In this observational, non-therapeutic pilot study, endometrial biopsies were taken after informed consent according to the Ethical Committee of the University of Heidelberg, Germany. A total of 27 women, at age 25 to 42 years, who were not on any hormonal treatments were included in this study. All women exhibited regular 28 ± 1 day cycles. Pipelle biopsies were taken in the mid-secretory phase on day 8-9 after the LH surge (LH +8/+9) from 11 women with RIF, 9 women with RSA, and 7 healthy woman. RIF was defined by failure to detect a positive serum hCG after three transfers of two or three good quality embryos created through in vitro fertilization. RSA was defined as the appearance of three or more spontaneous abortions of unknown reasons during the first trimester. None of the women with RIF or RSA had ever had a successful term pregnancy. Control women delivered healthy babies at term, with one woman having had a preterm delivery due to premature rupture of membranes. None of the women included in the study had known contributing factors for pregnancy failure, including thrombophilia (Factor V Leiden mutation, prothrombin mutation, antithrombin III, as well as protein C or S deficiency), antiphospholipid syndrome (anticardiolipin antibodies, lupus anticoagulans, ANAs), uterine anomalies detected by ambulatory hysteroscopy, polycystic ovarian syndrome, hormone abnormalities (thyroid, prolactin etc.) and other endocrinopathies (e.g., diabetes). On the day of biopsy blood samples were collected for determination of estradiol-17β (E2) and progesterone concentration in serum using a competitive immunoassay by the University of Heidelberg core facility. The LH surge was determined using a commercially available urine LH kit (Clear blue, WICK PHARMA/Procter & Gamble GMBH, Schwalbach am Taunus, Germany). Furthermore, histology of the biopsies typical for the secretory phase was confirmed by two individual researchers according to the modified Noyes' criteria [20].

### Tissue preparation

Immediately after the biopsies were performed, tissues were immersed in OCT, frozen in liquid nitrogen and stored at -80°C. Tissues were then sectioned at 8 μm, mounted onto SuperFrost Plus slides (Menzel GmbH & Co, Braunschweig, Germany), fixed with 100% acetone at 4°C for 10 min, and stored at -70°C before immunohistological procedure.

### Immunohistochemistry

Cell proliferation was determined based on immunolocalization of Ki67 protein (a marker of proliferating cells) and vascularisation was determined by immunolocalization of factor VIII (a marker of endothelial cells) and SMCA (a marker of pericytes and smooth muscle cells) as previously described [21,22]. Briefly, sections of cryopreserved endometrial tissues were rinsed several times in PBS containing Triton-X100 and were then treated for 20 min with blocking buffer [PBS containing normal goat serum (1-2%, v/v)] followed by treatment with primary antibody against Ki67 (1:100; mouse monoclonal; Vector Laboratories, Burlingame, CA, USA), factor VIII (1:100; rabbit polyclonal; Sigma, St. Louis, MO, USA) or SMCA (1:150; mouse monoclonal; Oncogene Research Products; San Diego, CA, USA) overnight at 4°C. Primary antibody was detected using a biotin-labeled secondary antibody (anti-mouse antibody for Ki67 and SMCA, and anti-rabbit antibody for factor VIII; Vector Laboratories) and the ABC kit (Vector Laboratories). For color development, Vector SG substrate kit (Vector Laboratories) was used. Sections stained for the presence of Ki67 were counterstained with fast red (Sigma) to visualize cell nuclei. Control sections were incubated with normal mouse IgG (4 μg/mL) or rabbit IgG (diluted 1:100) in place of primary antibody.

### Image analysis

Image analysis was performed as described in detail previously [22,23]. Endometrial images of randomly chosen areas (5-10 per uterine section/subject; 0.025 mm^2 ^per field) stained for Ki67, factor VIII or SMCA were taken at 400 × magnification, using the Eclipse E600 Nikon microscope and digital camera. Labeling index (LI; proportion of proliferating Ki67-postive cells out of the total cells per area), vascularity based on relative expression of factor VIII (occupied by endothelial cells) and SMCA (located in smooth muscle cells and pericytes) were determined by using computerized image analysis (Image-Pro Plus, version 5.0; Media Cybernatics, Houston, TX, USA). For factor VIII and SMCA, data are expressed as the total area that exhibited positive staining within tissue area.

### Statistical Analysis

Data were analyzed using the general linear model (GLM) procedures of SAS (SAS Inst. Inc. Cary, NC). When the F-test was significant (*P *< 0.05), differences among means were evaluated by using the least square means procedure [24]. Data are expressed as mean ± SEM.

## Results

The mean age of the women in the control group (30.3 ± 1.9 years) was similar to the women with RSA (34.6 ± 1.2 years) and the women with RIF (34.3 ± 0.8 years). E2 and progesterone serum concentration were similar in control, RSA and RIF (E2: 146 ± 21, 158 ± 25 and 128 ± 11 pg/ml; progesterone: 14.4 ± 4.3, 11.0 ± 1.7 and 10.2 ± 0.8 ng/ml), respectively.

**Figure 1:**
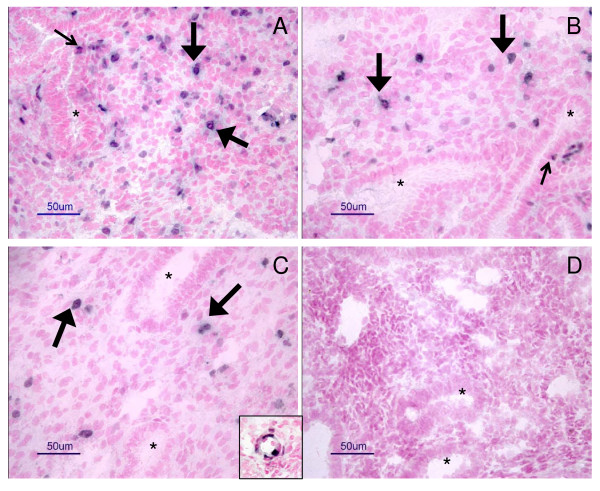
**Immunohistochemical staining of Ki67**. Representative micrographs of positive staining of Ki67 in human endometrium obtained from healthy women (A), and women with RSA (B) or RIF (C). Dark staining indicates proliferating cells, and pink staining indicates cell nuclei of non-proliferating cells. Note more proliferating cells in A than in B or C. Proliferating cells in endometrial glands are marked with small arrows in A and B, and in stromal tissue with large arrows in A, B and C. Insert in C demonstrated proliferating cells in a blood vessel. Endometrial glands are marked with stars (*). Control staining did not show any positive staining for Ki67 (D). Bar = 50 μm.

**Figure 2:**
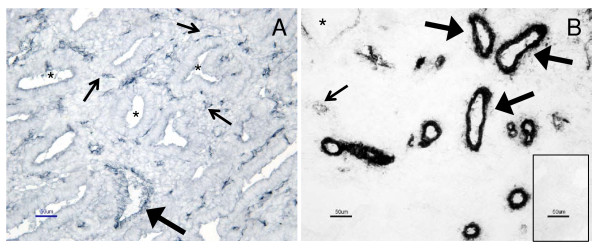
**Immunohistochemical staining of factor VIII and SMCA**. Representative micrographs of positive staining (dark color) of factor VIII (A) and smooth muscle cell actin (B) in human endometrium obtained from healthy women. Note positive staining in small (small arrows) and larger (large arrows) blood vessels. Endometrial glands are marked with stars (*). Control staining did not show any positive staining for factor VIII and SMCA (insert in B). Bar = 50 μm.

Ki67, factor VIII and SMCA were detected in uterine tissue sections in all groups (Fig. 1 and 2). Ki67 was detected in cells of uterine glands, stromal tissue and blood vessels (Fig. 1A-C), while factor VIII (Fig. 2A) and SMCA (Fig. 2B) were localized to blood vessels. Factor VIII was detected in small and larger blood vessels (Fig. 2A), and SMCA was detected mostly in the larger blood vessels, but also in some small blood vessels (Fig. 2B).

**Figure 3:**
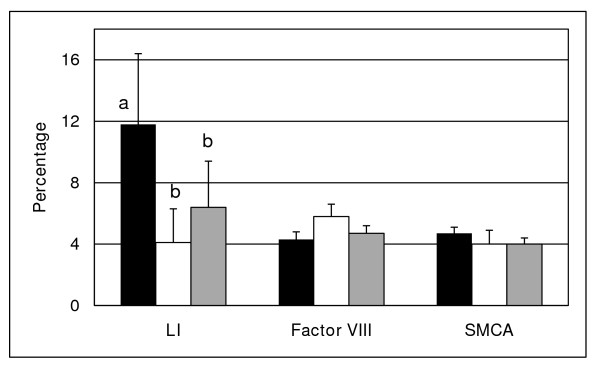
**Relative Expression of assessed factors in the control and study groups**. Labeling index (LI), expression of factor VIII and SMCA in endometrium of the control (black bars), RSA (open bars) and RIF (grey bars) groups. ^a,b^P < 0.0004 for LI.

The labeling index was greater (P < 0.0004) in endometrium of the control group compared to the endometrium of women with RSA or RIF, which were both similar (Fig. 3). The cell proliferation index of the different groups was not significantly correlated to the estrogen serum concentration (P = 0.369). The expression of factor VIII or SMCA in endometrium was similar in all three groups (Fig. 3).

## Discussion

The present data demonstrates that in two pathological groups, RSA and RIF cellular proliferation is less than in a control group. However, vascularisation of endometrium in these pathological groups was similar to the control group.

Although it is widely accepted that the quality of the embryo is the essential factor for successful pregnancy outcome, several studies have shown the importance of endometrial function in successful pregnancy outcome [5,14,25]. Numerous studies have focused on causes of implantation failures by examining embryo effects or endometrium at cellular and molecular levels [8,11,12,26,27]. The function of the endometrium is to allow implantation on one hand, but make sure that invasion of the uterine tissue is limited, as otherwise inadequate placentation occurs, including placenta accreta, increta, percreta [28]. Furthermore, endometrial cell proliferation and differentiation has to be tightly controlled [14], as progression to endometrial cancer can be seen when uninhibited proliferation takes place [29]. In the present study, inadequate endometrial growth marked by reduced cell proliferation in endometrium could contribute to reproductive failure. Although very few studies investigated endometrial cell proliferation and its regulatory mechanisms, decreased proliferation is found in endometrium at the time of menopausal transition in women [30], while in endometriosis, cellular proliferation in the human endometrium was enhanced around the time of implantation and during late secretory phase [31]. Furthermore, Mertens et al have described ongoing tissue proliferation of endometrial stromal cells via Ki67 expression in the secretory phase despite rising progesterone levels [32]. In agreement with our results, Lee and colleges [11] have indicated that defective cell growth, regulated by the differentially expressed genes controlling the cell cycle, is likely to contribute to implantation failure.

Numerous growth factors of uterine tissues including fibroblast growth factors, epidermal growth factors, vascular endothelial growth factor and others are involved in the control of endometrial growth and function [33,34]. In addition, estrogens and progesterone are also important regulators of endometrial function [15,35]. However, in our study as well as in other studies, concentration of E2 and progesterone were similar in normal and pathological conditions [36]. This suggests that inadequate expression of growth factor(s), rather than steroid hormones contributed to decrease the cell proliferation in reproductive failure cases. Nevertheless the effect of steroid hormones cannot be excluded, since a difference in distribution of nuclear and cytoplasmic progesterone receptors was found in women with early pregnancy loss compared to women with proven fertility [36]. In addition, the progesterone receptor is thought to play a role in the implantation defect of women with RIF [37]. Last but not least a reduction of estrogen receptor alpha at the time of implantation is described in women with RIF [10]. Therefore, further investigation is needed to evaluate the potential impact of the changes in hormone receptors on endometrial cell proliferation in women with reproductive failure.

Since cellular proliferation is reflected by tissue growth and development, endometrial thickness and/or volume is likely to depend on the rate of cell proliferation at specific reproductive stages. In fact, several studies have demonstrated a positive association between pregnancy rate and endometrial volume and/or thickness [38,39]. Thus, these observations are in agreement with our data showing reduced cell proliferation in reproductive failures. On the other hand, several studies did not observe any association between endometrial volume or endometrial thickness and pregnancy outcome [16,40]. These discrepancies are likely due to different timing and treatments applied before measurement of endometrial volume or thickness. Therefore, more detailed and uniformed studies should be performed to determine the role of endometrial cell proliferation, volume and thickness in reproductive failure.

In the present study, we demonstrated that vascularization of endometrium was similar for controls and women with reproductive failure, indicating a limited role of blood vessel function in implantation failure. In agreement to our study, Plaisier et al also showed no difference in vascularisation on the maternal side of implantation, namely decidualized endometrial stromal cells, in cases with missed abortions compared to controls, while they were able to demonstrated differences in receptors of growth factors in decidua parietalis and basalis at the time of missed abortion [41]. Although the importance of angiogenesis for implantation, and normal uterine and placental function is well recognized [16,42,43], the role of inadequate vascularization or blood flow in implantation failure has not been investigated in detail. Inconsistent results of impact of uterine blood flow in implantation failure measured by ultrasound technologies were obtained [16,17]. For example, using ultrasound techniques [44], reduced endometrial and subendometrial 'perfusion' has been demonstrated which suggests a reduced vascularity in women with unexplained subferitility, irrespective of E2 and progesterone levels. In contrast, Ng et al. [45,46] did not find any association between infertility and blood flow measured via ultrasound techniques. These latest results are in agreement with our studies, since we did not observe changes in blood vessel density in reproductive failure subjects. The discrepancies of results in studies cited above are likely due to different techniques (ultrasound vs. immunohistochemisty and image analysis), different population and/or study design used in these experiments. Since human studies are restricted in their design, the studies performed in human tissue are limited in their character, therefore we were only able to look at the intrinsic endometrial regulation of women with reproductive failure at the protein level. Thus, additional studies should be performed to determine mRNA and protein expression especially for genes regulating cell proliferation in endometrium from women with and without reproductive failure.

## Conclusion

In summary, we demonstrate a decrease in cell proliferation in endometrial tissues from the mid-secretory phase in women with reproductive failure compared to healthy controls. However, vascularisation in endometrium was similar for the examined groups. We therefore hypothesize that adequate endometrial growth is a critical factor contributing to the success of implantation, as well as further continuation of an established pregnancy. Our results help to better understand the underlying pathomechanism of reproductive failure and may contribute to a discovery of marker(s) which can be used to predict successful vs. non-successful pregnancy.

## List of abbreviations

ANA: antinuclear antibodies; ART: assisted reproduction technique; mRNA: messenger ribonucleic acid; C/EBPbeta: CCAAT/enhancer binding protein (C/EBP), beta; CRABP2: cellular retinoic acid binding protein 2; E2: estradiol-17β; hCG: human choriogonadotropin; IgG: Immunglobulin G; Ki67: proliferation marker; LH: luteinising hormone; PBS: phosphate buffered saline; RSA: recurrent spontaneous abortions; RIF: recurrent implantation failure; SG: silver grain; SMCA: smooth muscle cell actin.

## Competing interests

The authors declare that they have no competing interests.

## Authors' contributions

AG participated in designing the study, coordinated tissue collection, evaluated results and participated in writing the manuscript and was responsible for intellectual content of the study; MvW participated in patient selection, consenting the patients and tissue collection; JJ participated in tissue collection and preparation for immunohistochemistry; TS participated in patient selection and in writing of the manuscript; TS performed image analysis and prepared data for statistical analysis; ATGB participated in designing of the study, evaluated results and participated in writing the manuscript, coordinated and supervised immunohistochemical staining and image analysis. All authors read and approved the final manuscript.
